# Evaluation of SNaPshot and Sanger sequencing for the detection of *KRAS* and *NRAS* mutations in a sample of Venezuelan patients with colorectal cancer

**DOI:** 10.3332/ecancer.2024.1797

**Published:** 2024-11-13

**Authors:** Daniela Rodríguez-Carrascal, Rafael Puche, Mary Acosta, Carlos Darío Ramírez

**Affiliations:** Instituto Venezolano de Investigaciones Científicas (IVIC), Unidad de Estudios Genéticos y Forenses (UEGF), Caracas 1020, República Bolivariana de Venezuela

**Keywords:** KRAS, NRAS, SNaPshot, Sanger sequencing, colorectal cancer, Venezuela

## Abstract

Colorectal cancer (CRC) is the third most commonly occurring cancer in men and the second most commonly occurring cancer in women. The epidermal growth factor receptor (EGFR) is relevant in the development and progression of CRC, because it is part of multiple signaling pathways involved in processes of the cell cycle, their malfunction causes dysregulation and subsequently carcinogenesis. Consequently, therapies were developed with anti-EGFR monoclonal antibodies (MAbs) that improve the survival of patients with CRC. However, mutations in the oncogenes Kirsten rat sarcoma (KRAS) and Neuroblastoma RAS (NRAS), modulators of the EGFR signaling pathway (downstream) activate a pathway independent in which such drugs have no effect. Patients with these mutations have a low response to MAb therapies. In this research, the SNaPshot sequencing method was used for the first time in Venezuela for the diagnosis of mutations in exon 2 of the KRAS and NRAS genes, from DNA extracted from tumor tissue samples fixed with formalin and included in paraffin (FFPE) and was compared with Sanger’s method to determine the specificity and sensitivity, in the detection of mutations in the KRAS and NRAS genes. Of the 33 samples analysed, 27.3% presented mutations in KRAS and 15.1% in NRAS. With the obtained, it was carried out for the first time in the country the assignment of the geographical distribution of the polymorphisms found in these genes. The mutational status of the KRAS and NRAS genes showed no relationship statistically significant with clinical-histopathological variables. For this study, the SNaPshot method showed greater accuracy, sensitivity and specificity in the detection of single nucleotide polymorphisms than the Sanger method.

## Introduction

Cancer is a disease with a high mortality rate worldwide. According to data from the World Health Organization in its latest Global Observatory report of cancer by the year 2022 (GLOBOCAN 2022) [1]. There were close to 20 million new cases of cancer in the year 2022 (including nonmelanoma skin cancers (NMSCs)) alongside 9.7 million deaths from cancer (including NMSC). The estimates suggest that approximately 1 in 5 men or women develop cancer in a lifetime, whereas around 1 in 9 men and 1 in 12 women die from it. Lung cancer was the most frequently diagnosed cancer in 2022, responsible for almost 2.5 million new cases, or one in eight cancers worldwide (12.4% of all cancers globally), followed by cancers of the female breast (11.6%), colorectum (9.6%), prostate (7.3%) and stomach (4.9%). Lung cancer was also the leading cause of cancer death, with an estimated 1.8 million deaths (18.7%), followed by colorectal (9.3%), liver (7.8%), female breast (6.9%) and stomach (6.8%) cancers. Breast cancer and lung cancer were the most frequent cancers in women and men, respectively (both cases and deaths).

The most common gene mutations in colorectal cancer (CRC) are the adenomatous polyposis coli gene mutation which accounts for around 30%–70% of the sporadic adenomas and sporadic CRCs. *BRAF* mutations are identified in around 4% of the microsatellite low (MSI-low) and 40% in MSI-high CRC tissues. *TP53* mutation accounts for 43% of all the CRC cases. Around 40%–52% of CRC cases are characterised by a mutation in the Kirsten rat sarcoma (*KRAS)* gene. Mutations also occur in other genes such as *SMAD4, BRIP1, CHEK2, MUTYH, HNF1A* and *XPC*. Among all these gene mutations, *KRAS* has been considered as one of the ‘undruggable’ targets in cancer treatment [3].

In most pathology laboratories, direct sequencing, i.e., polymerase chain reaction (PCR) followed by dideoxy sequencing (Sanger), is considered the gold standard for* KRAS* mutation detection. However, this technique is not only laborious and time consuming, but sensitivity plays an important role. To reliably test a sample, at least 20% to 30% of tumor cells are needed. To date, there are several alternative assays available for (*KRAS*) mutation detection, including ‘homebrew’ assays, such as high-resolution melting curve analysis, pyrosequencing, single nucleotide primer extension assay and allele-specific real-time PCR,1 and commercially available assays, such as reverse hybridization test KRAS StripAssay (Vienna Labs, Vienna, Austria) and real-time PCR–based TheraScreen (Roche Diagnostics, Almere, the Netherlands); all these assays greatly differ in sensitivity, specificity, DNA input, time to results, hands-on time, flexibility, workload and costs. The single nucleotide primer extension (SNaPshot) assay is a home-brew, flexible assay, which might be easily extendable to other biomarkers, whereas from the commercially available assays, the KRAS StripAssay claims to be fast and very sensitive [4].

In Venezuela, according to the projections of the Venezuelan Anticancer Society (SAV) [2], the estimates made for cancer without distinction of cancer type for women, they foresee an increase for both mortality and incidence. As for mortality, an estimated 25% growth in the number of deaths in relation to the year base, 2016, this implies that 23 more deaths are expected per 100,000 women. For the incidence, the increase over the base year, 2019, is expected at 7% of new cases, that is, for every 100,000 women, 12 more cancer cases are expected to increase by 2023. For men, an increase is expected in both the number of deaths (26.55%) and new cases (9.23%), although it is expected to increase 8 more deaths and 7 more cases for every 100,000 men. In the case of both genders, an increase is expected in mortality and incidence. For mortality is forecast during the year 2023 an increase of 24.74%, corresponds to 16 more deaths per 100,000 inhabitants, and for the incidence, the increase is 10.72%, that is, 21 more cases per 100,000 inhabitants.

## Materials and methods

### Sample selection

Samples were selected from 391 formalin embedded in paraffin tumor (FFPE) plates, which were received between 2008 and 2015 at the Genetic and Forensic Studies Unit (UEGF) of the Venezuelan Institute of Scientific Research (IVIC), according to the variables of interest. The selection of the samples was carried out randomly, taking into account that for each sample the histories provided all the data of the clinical-histopathological variables of interest. Thirty-three fixed in were selected from patients with confirmed diagnosis were analysed of CRC, five of them were used in the standardization of some of the techniques molecular used. All patients provided a history form clinical and histopathological information of the tumor, together with an informed consent signed and verified by the IVIC Bioethics Committee. The description and classification of the patients according to these variables were used for the creation of a database, made with the subsequent objective of relating such data with the status (not mutated (WT) or mutated) of the variants that are detected in the *RAS* genes analysed.

The clinical-histopathological variables taken into account for the classification of data of the present study were: 1. Gender: male and female. 2. Age (ranges): under 45, from 45 to 59, from 60 to 75 and over 75 years. 3. Tumor localization: ascending colon, transverse colon, descending colon, sigmoid and rectum. 4. Grade of tumor differentiation: well, differentiated (grade I), moderately differentiated (grade II) and poorly differentiated (grade III). 5. Histological pattern: adenocarcinoma; adenocarcinoma *in situ*; serrated or adenomatous adenocarcinoma; medullary carcinoma; mucinous or colloid adenocarcinoma; signet ring cell carcinoma; squamous cell or epidermoid carcinoma; adeno-squamous carcinoma; and neuroendocrine carcinoma (modified from) [6]. In addition, the American Joint Committee on Cancer (AJCC) system tumor, lymph node, metastasis (TNM) system was used to perform tumor staging for each case studied [7]. After classifying the patients according to these parameters, the staging was carried out according to the prognostic groups according to García [8], from 0 to IVB.

### Preparation of the samples

Initially, the samples were prepared as follows: the FFPE tissue blocks were subjected to a dewaxing process with xylol, performing two dips of 5 minutes each. Then, the xylol was removed with successive washes at decreasing concentrations of ethanol (100% and 95%), and then the open tubes were placed in the incubator at 37º for 15 minutes, to evaporate the ethanol residues.

### DNA extractions

DNA extraction by the silica column method was performed using the commercial GeneAid (GSync DNA extraction kit). The alternative method used for the extraction of DNA from tissues was the Salting-out method, which consists of the precipitation of proteins by dehydration, using a saturated NaCl solution [9]. To determine the amount of DNA and the yield of the extraction methods used, a spectrometric quantification was performed using a NanoDrop 2000 spectrophotometer (Thermo Fisher Scientific) and the associated program NanoDrop 2000/2000c v1.6 compatible with Windows 10. All samples were analysed for verification of the amplification products by electrophoresis in a 1% agarose gel.

### Purification of the amplified products

In this step, excess primers and nucleotides that could interfere with the subsequent reaction for sequencing were removed using the enzymatic reaction of ExoSAP-IT; 2 µl of ExoSAP-IT was added to 5 µl of the amplified products, mixed and taken to the thermal cycler with the following program: 45.37ºC, 15.85ºC. However, when performing the standardization of the SNaPshot sequencing method, it was noticed that the products purified with ExoSAP-IT generated readings of unspecific peaks that prevented the visualization of a correct result. Therefore, a test was carried out using the magnetic bead purification method (MagneSil GREEN^®^). To obtain purified products and eliminate the contaminating residues of reagents used in the previous experimental steps, the magnetic bead purification method was used [10].

### DNA sequencing by the SNaPshot method

The detection of mutations in exon 2 of the *KRAS* and Neuroblastoma RAS (*NRAS)* genes was carried out by the SNaPshot sequencing method, which detects mutations of several single nucleotide polymorphism (SNP) markers simultaneously [11, 12]. The selection of extension primers was made according to the specific positions for the possible mutations in exon 2 of the *KRAS* and *NRAS* genes [13, 14].

A Genetic Analyser sequencer (3130XL Applied Biosystems) and POP-4 polymer with the Buffer TAP Buffer solution (tris-hydroxymethyl-methylamino) at pH 8.0. After electrophoresis and fluorescence detection, each peak obtained corresponded to one base of the study codon, and the alleles of a single marker appeared as peaks of different colors, of similar sizes on the electropherogram graph [12].

### Statistical analysis

After obtaining all the data from the laboratory analysis, for all the samples, the statistical analysis was carried out through the R programming language (R Core Team, 2020; version 3.6.3), in conjunction with the RStudio interactive development environment (version 1.2.533) [15].

### Calculation of mutation frequencies

With the results of the laboratory analysis of the sequences obtained by the SNaPshot sequencing method, a direct count was performed and the patients were classified according to the status of the mutations in the genes (WT or mutated). In addition, each of the possible SNPS was determined. Finally, the geographical distribution of the analysed samples was assigned (according to the data collected in the medical records), in addition to the polymorphisms that they presented in the study of the *KRAS* and *NRAS* genes.

### Relationship between variables

The association between the mutational status of the studied genes with the clinical-histopathological variables was studied. For this, the database made at the beginning of the study was used. Each of these variables was evaluated against the mutational state of the *KRAS* and *NRAS* genes, respectively, through the Fisher Exact Test with the use of contingency tables, the null hypothesis that the mutational state is independent of each of these variables was tested, with a 95% confidence limit.

### Comparison of the SNaPshot method with Sanger technique

The results of the analysis of the KRAS gene in 33 samples from patients with CRC by the SNaPshot technique were compared with previously obtained results for the same samples using the Sanger sequencing method. The results of the method of Sanger were obtained before the start of this project, in the routine diagnosis of mutations in the *KRAS* gene in the IVIC-UEGF [16]. Additionally, sensitivity and specificity tests were performed on contingency tables (2 × 2) for both methods.

## Results

### Selection of samples and classification of variables

The creation of a database of 391 samples from CRC patients received at the IVIC UEGF allowed the rapid randomised selection of the 33 samples selected for this study, with all the samples met the requirements of having the complete clinical history, including the anatomopathological information and the sequencing tests by the Sanger method. They were classified according to the clinical-histopathological variables considered above and summarised in Table 1.

Of the 33 samples analysed, 18 belonged to female patients (54.5%) and 15 to male patients (45.5%). These in turn were divided into different age ranges, most often in the range of 45 to 59 years in female patients, and in the case of male patients, the age ranges were most often from 45 to 59 and from 60 to 74 years interchangeably. On the other hand, the location of the primary tumor of the patients in the cohort was diverse. However, they were most often located in the ascending colon (38%), followed by rectum (24%), transverse colon (15%), sigmoid colon (12%) and descending colon (12%). It was also observed that the majority of these tumors had a low degree of differentiation (78.8%), of which 14 (42.4%) were grade I and 12 (36.4%) were grade II; the rest (7 samples, 21.2%) had a high degree of differentiation or grade III. On the other hand, the histological pattern observed was also diverse, most often adenocarcinoma, followed by mucinous adenocarcinoma. In addition, the patients were classified in the TNM system and the corresponding stage was assigned as shown in Table 2, in which it can be seen that most of the patients presented localised metastasis in one organ, that is, they were in stage IVA (51.5%).

### SNaPshot sequencing method

**Figure 1.**
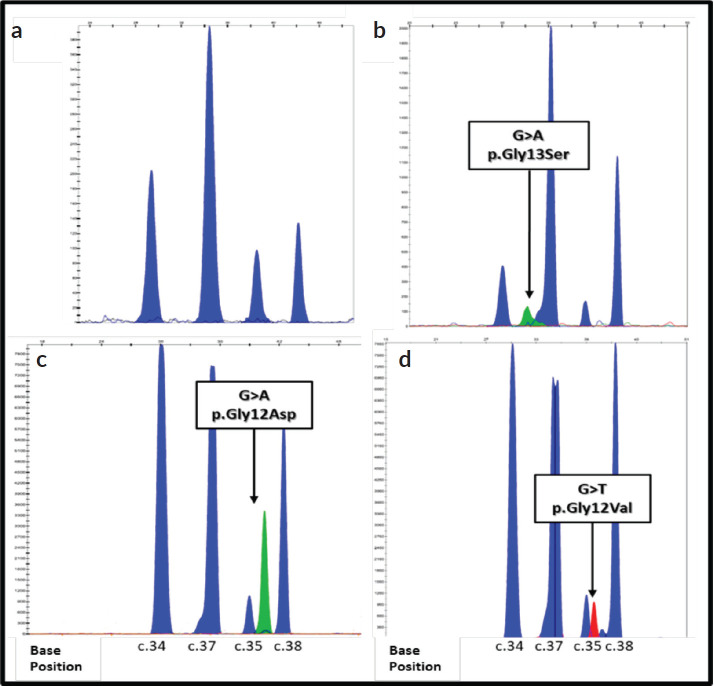
Electropherograms of the detection of mutations in the KRAS gene using the SNaPshot method. Each peak corresponds to an extended primer specific. The positions of the nucleotides are indicated at the bottom of the figure. The arrows indicate the location of each mutation. The bases are represented by the following colors: A: green; C: black; G: blue; T: red. (a–d): Show results of genotyping obtained using DNA extracted from FFPE colorectal tumor tissue from samples 12, 31, 32 and 28.

The validation of the primers was carried out, using the commercial SNaPshot Primer Focus kit, with which it was possible to observe that the primers designed for the detection of mutations in the *KRAS* gene have different mobilities during electrophoresis, which allows them to be distinguished in a multiple run as is done for the SNaPshot reaction. However, both the primers designed for positions 34 and 35, and the primers designed for positions 37 and 38, of the *NRAS* gene, have very similar mobilities in the electrophoresis (Figure 1), so their spectra may possibly overlap in simultaneous runs. According to this result, the multiplex SNaPshot method had to be performed separately in two different systems with the extension primers for the *NRAS* gene, one for positions 34 and 38 (N1) and another for positions 35 and 37 (N2), and thus ensure the correct separation of the fragments and their visualization during electrophoresis (data not shown).

**Figure 2.**
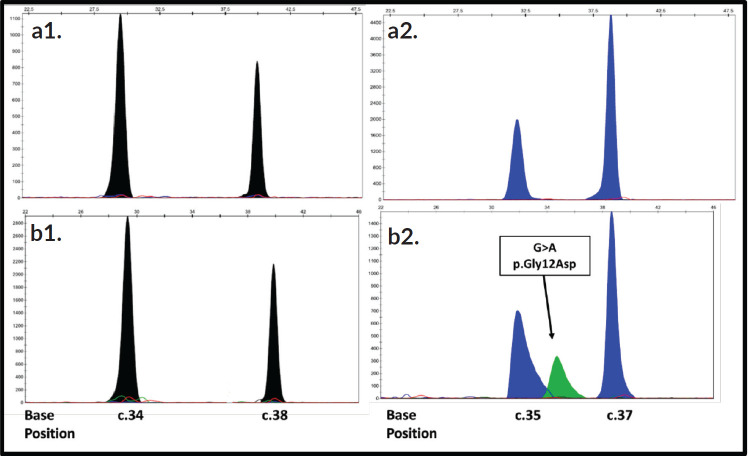
Electropherograms of the detection of mutations in the NRAS gene using the SNaPshot method. The primers 34 and 38 present are aligned in reverse direction so the reading obtained is from the extended base in the string 3′≥5′. The positions of the nucleotides are indicated at the bottom of the figure. The arrows indicate the location of each mutation. The bases are represented by the following colors: A: green; C: black; G: blue; T: red. Electropherograms (A1 and A2): Correspond to a WT sample (sample #4), while electropherograms (B1 and B2): represent a mutated sample for NRAS (sample #33).

The electropherogram of Figure 1A, belongs to a WT sample (sample #12) of the analysis of the *KRAS* gene, the four blue peaks (G) indicate the four known SNPs of the codons 12 and 13 of exon 2. In the samples in the mutated state, an additional peak is observed that overlaps or juxtaposes with any of the peaks of the electropherogram WT, indicating the presence of a mutation in the heterozygous state. Figure 1B (sample #31), two peaks at the C.37 nucleotide showing the ‘G’ and ‘A’ genotype indicate the c.37 mutation G>A which results in the P. Gly13Ser change in the KRAS protein. Figure 2 (sample #32), shows the mutation c .35 G>A, p. Gly12Asp; while Figure 2 (sample #28), presents the change c.35 G>T P. Gly12Val.

The electropherograms of Figure 2: A1 and A2, belong to a sample WT (sample #4) from the *NRAS* gene analysis, the two blue peaks indicate the presence of G at positions 35 and 37 of exon 2, while the two black peaks are due to the presence of C at positions 34 and 38 of exon 2, this is because the extension primers used had the reverse sense (3′≥5′), therefore these bases must be interpreted instead of C as G which would be its peer in the sense string 5′≥′3. Figure 2: B1 and B2, represent a sample in a mutated state (sample #33) for the *NRAS* gene, in which an additional peak is observed that is juxtaposed to the WT peak of position 35, indicating the presence of the heterozygous mutation C.35 G>A which results in the p. Gly12Asp change in the NRAS protein.

A description of the results obtained is presented in Table 3 with the samples analysed in this study. First of all, the sample code is indicated, then the result obtained for the analysis of the *KRAS* gene and the *NRAS* gene, depending on whether it was WT or mutated, the latter indicating the expected amino acid change in the protein.

### Frequency of mutations

The laboratory analysis revealed that, of the 33 samples from the tumor biopsies, 9 (27.3%) had mutations in the *KRAS* gene and 5 (15.1%) in the *NRAS* gene, in the genetic positions studied. Of the 9 cases of *KRAS* mutations, 7 cases (21.2%) of codon 12 mutation were found, including 3 cases (9.1%) of the p. Gly12Asp change, 2 cases (6.1%) of p. Gly12Arg and 2 cases (6.1%) of p. Gly12Val. In addition, 2 cases (6.1%) of codon 13 mutation were found, all carrying the p. Gly13Ser change (Table 4).

On the other hand, of the mutations detected in the *NRAS* analysis, 5 cases (15.1%) belonged to codon 12 mutations, including 3 cases (9.1%) of the p. Gly12Asp change and 1 case (3.0%) of p. Gly12Cys. Also, 1 case (3.0%) of codon 13 mutation was found, with the change p. Gly13Ala (Table 5).

Probably due to the low frequency, no mutations were detected at position 38 belonging to codon 13 of *KRAS* exon 2, nor at position 37 of codon 13 of the gene *NRAS*, in our cohort.

**Figure 3.**
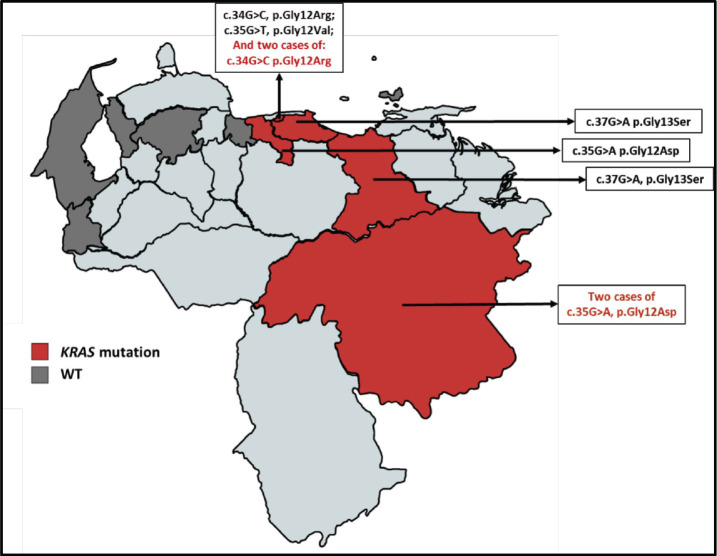
Geographical distribution of the KRAS gene mutational status for the sample selected. The red color denotes the presence of patients carrying a mutation in KRAS and the dark gray color refers to the presence of patients with KRAS WT.

**Figure 4.**
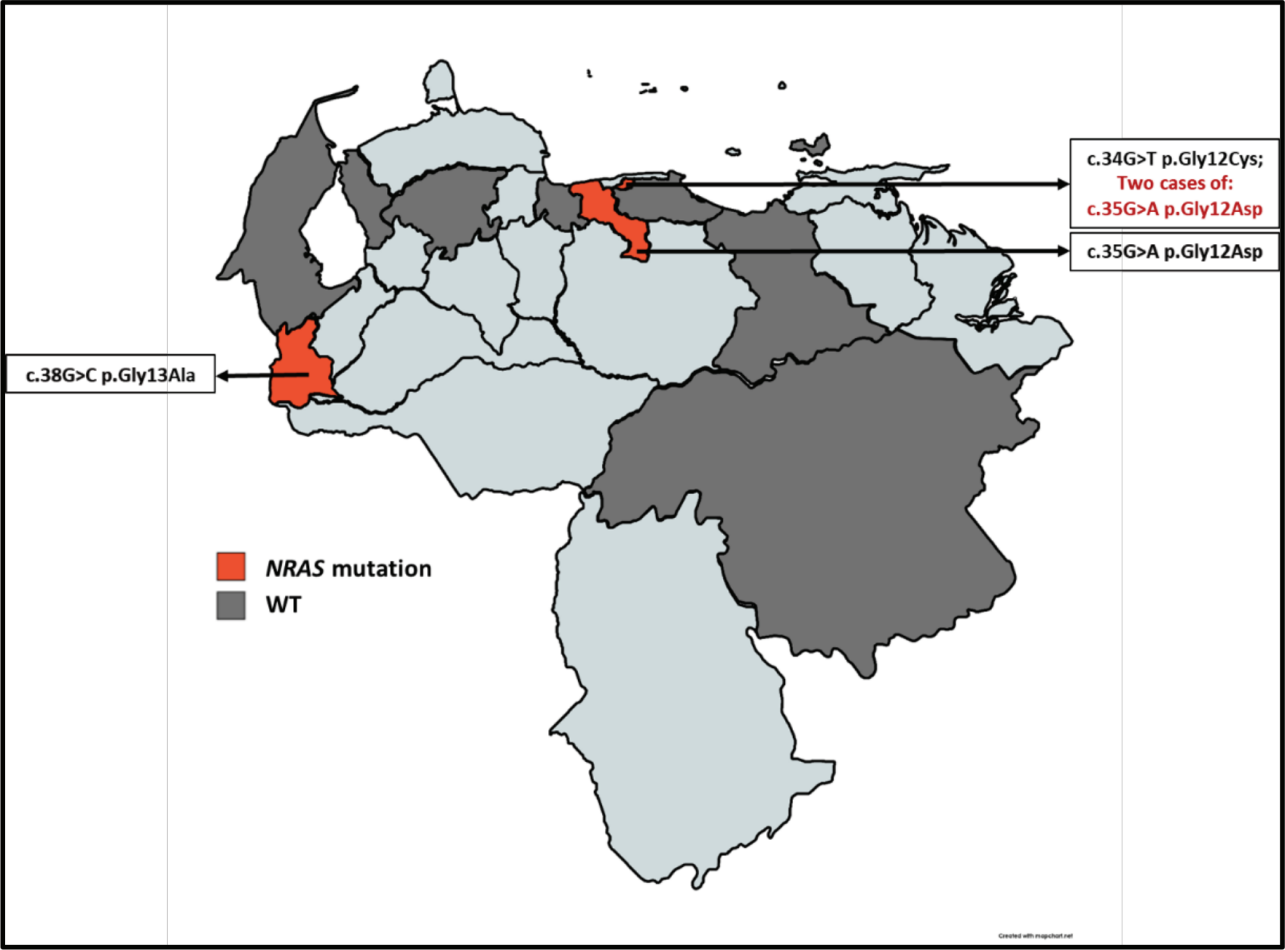
Geographical distribution of the NRAS gene mutational status for the sample selected. The red color denotes the presence of patients carrying a mutation in NRAS and the dark gray color refers to the presence of patients with NRAS WT.

Subsequently, the assignment by the geographical distribution of the samples was carried out and analysed, in which we initially denote the number of samples obtained by the state throughout the country. Then the geographical distribution was made according to the state mutational of the *KRAS* (Figure 3) and *NRAS* (Figure 4) genes, which also denoted which mutations were found according to the origin of the patient.

## Relationship between variables

The two-tailed Fisher exact test was used to test the null hypothesis that the variable of the mutational state of the KRAS or NRAS gene was independent of the following clinical-histopathological variables: sex, age, tumor location, stage and degree of tumor differentiation. The confidence interval used was 95%. However, the obtained *p*-values indicate that there is insufficient evidence to reject the null hypothesis that the mutational status variable and the clinical-histopathological variables are independent.

### Comparison of the SNaPshot method with Sanger technique

The results of the diagnosis of mutations in the gene were compared *KRAS* through the SNaPshot method and the Sanger method, as shown to continuation.

From the table (Table 6), it should be noted that the diagnosis of mutations in the gen *KRAS* by SNaPshot method showed 91% (*n* = 30) analytical accuracy with respect to the Sanger sequencing method. In the other 6% (*n* = 2), the method of SNaPshot managed to detect mutations that were not identified by Sanger’s method. In the remaining 3% (*n* = 1), no mutations were detected with the SNaPshot method if they were previously reported by Sanger’s method. Based on these results, sensitivity and specificity tests were carried out in contingency tables (2 × 2) for the SNaPshot method and the Sanger method, the results obtained are presented in Table 7.

## Discussion

CRC is a heterogeneous disease at the cellular and molecular levels. KRAS is a commonly mutated oncogene in CRC, with mutations in approximately 40% of all CRC cases; its mutations result in constitutive activation of the KRAS protein, which acts as a molecular switch to persistently stimulate downstream signaling pathways, including cell proliferation and survival, thereby leading to tumorigenesis. Patients whose CRC harbors *KRAS* mutations have a dismal prognosis. A study conducted on three continents reports the frequency of mutations in the gene *KRAS* of 36%, similar to the frequency reported in Europe (40%), but higher than in Asia (22%) [35]. In this study, Venezuela is profiled with a high rate of mutated *KRAS* gene of (33%) [17]. On the other hand, a review on the pharmacogenomics of the *KRAS* gene was recently published, in which the authors explain the incidence of the different mutations of this gene for 7 types of cancer, revealing an approximate mutation frequency of 45% in the United States and 49% in China of CRC cases. Additionally, they reported that Gly12Asp and Gly12Val were the two mutated alleles of more common *KRAS* in the CRC [19].

Several studies carried out in Venezuela (monocentrics), on the frequency of mutations in the *RAS* genes, report different findings. In an analysis carried out in the city of Maracaibo (west of the country), mutations in codons 12 and 13 of the *KRAS* oncogene were found in 23.33% of patients, of these 28.57% in codon 12, and in codon 13 57.14% were found [25]. Another investigation carried out in Caracas 20.00% reported mutations in *KRAS*, of which 85.71% had mutations in codon 12 and 14.28% in codon 13 [26]. More recently, an analysis in the same city reported a frequency of 13.15% for the *KRAS* gene and 2.6% for the *NRAS* gene [18].

With the selected samples in our study (*n* = 33), a database was created with the clinical-histopathological variables, which showed a high heterogeneity between samples. From this database, it could be noted that the majority of patients belonged to the female sex (*n* = 18, 55%), despite the fact that according to data from the SAV this type of cancer occupies the fourth place of incidence in women and the third in men. On the other hand, it found that the largest number of samples of female patients had ages between the ages of 45–59, as opposed to male patients for which a higher frequency was obtained in ages ranging from both 45–59 years and of 60–74 years, these data agree with those reported by the SAV [20].

Among the variables analysed, the degree of differentiation of the tumor tissue, also turned out to be of great interest, since in the study cohort most of the samples they presented a low degree of differentiation (*n* = 26; 78.8%), these cases were represented by the well-differentiated types (*n* = 14; 42.4%) and moderately differentiated (*n* = 12; 36.4%). The low degree of tumor differentiation indicates that the cell tumors have a certain similarity to normal cells and a replication capacity slow. Consequently, tumors with a low degree of differentiation are less aggressive (AJCC) [21]. On the other hand, the histological pattern predominant was adenocarcinoma, followed by its derived variants the adenocarcinoma mucinous and serrate adenocarcinoma, concordant with the literature [22, 23]. The samples were classified and staged with the TNM system showing wide variability (Table 2), it could be observed that 51.5% of the patients were in stage IV, that is, they had metastases in a distant organ of the colon and/or rectum, which represents a state of greater severity of the disease. According to previous reports, commonly only 25% of patients with CRC had metastases at the time of initial diagnosis [8, 24].

The frequency of *KRAS* gene mutations in this study turned out to be 27.3%, despite those lower frequencies had previously been reported in Venezuela [16, 18, 25, 26]. Now, to compare with the data on frequencies of mutations in the *KRAS* gene reported in different countries or at the continental level by other authors, the data reported in our country including the present work continues to be below that reported by various authors [17, 19]. Mutations were found to be more frequency at codon 12. First of all, Gly12Asp, followed by Gly12Val and Gly12Arg, this distribution of the different types of mutations observed, resembles the reported in the literature [19]. Regarding the analysis of the *NRAS* gene, a mutation frequency of 15.1% was obtained, quite high in relation to that previously reported both for the Venezuelan population in which this frequency varied between 1.6% and 2.6% of patients, and in other parts of the world where alterations of this gene appeared in approximately 3% or 5% of cases of CRC, and whose meaning should be studied further [27, 28]. Additionally, in this work, the Gly12Asp variant derived from the G>A change at position 35 of codon 12 was the most frequent. Like other studies, the alterations were found most frequently in codon 12, other authors also reported mutations in codon 61 as frequent [16, 29, 30].

With the data of origin of the patients of the study cohort, the allocation of the geographical distribution as shown in Figures 3 and 4. The distribution of the sample covered 9 states of Venezuela: Capital District, Bolívar, Aragua, Anzoátegui, Lara, Nueva Esparta, Miranda, Táchira and Zulia. It was obtained the highest incidence is in the Capital District, followed by Bolívar and Aragua. According to data from the SAV, Distrito Capital occupies the second place in incidence in patients with CRC [20]. At the same time, it was possible to realise the Venezuelan distribution of the polymorphisms found for the *KRAS* and *NRAS* genes, which had not been previously reported by other authors. The mutated *KRAS* genotypes, were located in 5 of the 9 states (Figure 3) of the analysed cohort (Capital District, Bolívar, Aragua, Miranda and Anzoátegui), while only 3 of these 9 states (Figure 4) presented mutated *NRAS* genotypes (District Capital, Aragua and Tachira); for both cases, a higher incidence was found in the District Capital.

Fisher’s exact test showed no existence of an association between the mutational status of *KRAS* (WT or mutated) and the following clinical-histopathological variables: sex, age, tumor location, histological pattern, stage (TNM) and degree of tumor differentiation; because no statistical evidence was observed significant in order to reject the null hypothesis that the mutational state results independent of these variables (*p* > 0.05). The same happened in the case of the *NRAS* gene (test Fisher’s exact, *p* > 0.05). Several previous studies also found no association between the *KRAS/NRAS* mutational status with age, sex and tumor localization [31–33]. In contrast, Sanchez-Ibarra *et al* [30] by studying a cohort of 500 Mexican patients with CRC metastatic obtained a statistically significant association between the mutational state of *KRAS* and *BRAF* (*p* = 0.0414 and *p* = 0.0065, respectively) and the location of the tumor in the right colon; in contrast to the *NRAS* gene where the association was not statistically significant; in addition, the authors developed an analysis of t-SNE and Artificial Neural Network which showed systematic associations between tumor location, age, city of origin, the histological subtype and the histological grade versus the mutational state of *KRAS* [30]. Also, the mutational state of *KRAS* has been associated with a histological pattern of classical papillary and serrate adenocarcinoma [34]. With the above, one might think that the sample size used in this study is not sufficient to demonstrate the existence of an association between the genetic profile of *KRAS/NRAS* and the clinical-histopathological variables taken into consideration.

The comparative analysis of the sequencing techniques obtained a 91% analytical accuracy in the diagnosis of mutations in the *KRAS* gene by the SNaPshot versus the Sanger method previously called the ‘Gold Standard of Diagnosis’ (Table 6). In other research was previously conducted this comparative analysis of the SNaPshot method versus the reference next-generation sequencing technique and a 99% accuracy was obtained, while at comparing SNaPshot with Sanger’s method found 100% accuracy [5]. However, in the comparison, it should be taken into account that the results obtained through these analytical methods depend largely on the quality of the starting material and may vary because the tumor samples are very heterogeneous according to the fixation conditions, type of biopsy, surgical piece and neoadjuvant treatment, so a more accurate method might depend on the type of analysed sample [14]. However, the tests of sensitivity and specificity, both for the SNaPshot method and for the Sanger, in order to have other comparative parameters between the two methods and for this analysis the SNaPshot method provided a higher sensitivity and specificity in detecting the mutations present in exon 2 (codons 12 and 13) of *KRAS*, compared to Sanger’s method. This confirms the hypothesis raised at the beginning of this study and what was previously published by other authors, that the SNaPshot method may be more accurate, sensitive and specific in the detection of PNS than Sanger’s method [4, 5, 13, 14]. Therefore, there is a probability that the difference between the mutational frequencies of the *KRAS* and *NRAS* genes previously reported in Venezuela [16, 18, 25, 26] and those reported in the present work, are partly due to these authors used Sanger’s method and here we see that the same probably is not the most suitable for the diagnosis of this type of genetic alterations.

The SNaPshot method is more sensitive and accessible for the detection of SNPs compared to other molecular methods used in other studies [36]. Recently (2023), Jin *et al* [39] used Sanger and Snapshot sequencing to determine the association between *KRAS* gene polymorphisms and genetic susceptibility to breast cancer in a Chinese population [39]. This approach has also been used in studies for the detection of mutations in patients with non-small-cell lung cancer [40].

To our knowledge, the SNaPshot sequencing method is used for the first time in Venezuela for this type of diagnosis, in order to determine whether estimates previously made in the country on the frequency of mutations in the *KRAS* and *NRAS* genes showed differences with respect to other parts of the world, due to the low sensitivity of the previously used tools or are really the reflection of the genetic frequency and the structure of the Venezuelan population. It is important to add that this study was conducted without knowing the percentage of tumor tissue present in FFPE biopsies, although it is recommended to perform this analysis prior to the experimental treatment to ensure that the DNA is extracted from the tumor tissue (of interest) and not the healthy tissue, which could represent a margin of error [37, 38]. This percentage determination analysis of tumor tissue was not performed in the biopsies of the analysed samples, due to the high financial cost it represented for this research project, but it is advised its inclusion in subsequent works. The SNaPshot technique meets all the necessary conditions for the detection of mutations in the *KRAS* and *NRAS* genes in patients with CRC, especially for middle- or low-income countries that may not have access to new sequencing technologies, since it allows several SNPs to be detected in a single reaction, decreasing costs and execution times, in addition, it is sensitive with low amounts of DNA, even degraded DNA, since it is effective with short length sequences [50-80pb] [5, 39].

## Conclusion

The SNaPshot technique meets all the necessary conditions for the detection of mutations in the *KRAS* and *NRAS* genes in patients with CRC, as it allows to detection of several SNPs in a single reaction decreasing costs and execution times, in addition to is sensitive with low amounts of DNA, even degraded DNA, as it is effective with short length sequences [50-80pb] [5], but the same has never been realised for this type of diagnosis in Venezuela patients. In this retrospective study, we carried out a characterization of the mutations in exon 2 of the *KRAS* and *NRAS* genes in a selected sample of Venezuelan patients with CRC through the SNaPshot method and compared the results with the Sanger sequencing technique. We have corroborated in this study that the SNaPshot method provides a higher accuracy analytical, sensitivity and specificity in the detection of mutations present in exon 2 (codons 12 and 13) of *KRAS* and *NRAS*, compared to Sanger’s method.

## Conflicts of interest

None.

## Figures and Tables

**Table 1.** Clinical and histopathological data of the samples selected in the study.

Code	Age	Gender	Location		Grading	Histological pattern
1	60	F	Colon	Sigmoid	2	Adenocarcinoma
2	52	F	Rectum		1	Adenocarcinoma
3	84	F	Colon	Transverse	2	Medullary carcinoma
4	34	M	Rectum		1	Mucinous adenocarcinoma
5	60	F	Colon	Sigmoid	2	Adenocarcinoma
6	53	M	Rectum		2	Adenocarcinoma
7	53	M	Colon	Descending	2	Mucinous adenocarcinoma
8	67	F	Colon	Transverse	2	Adenocarcinoma
9	56	F	Rectum		1	Adenocarcinoma
10	58	F	Colon	Ascending	1	Serrated adenocarcinoma
11	69	M	Colon	Ascending	2	Adenocarcinoma
12	45	M	Colon	Sigmoid	1	Adenocarcinoma
13	71	F	Colon	Ascending	1	Mucinous adenocarcinoma
14	78	M	Colon	Descending	1	Adenocarcinoma
15	55	M	Colon	Ascending	1	Medullary carcinoma
16	76	M	Colon	Ascending	2	Adenocarcinoma
17	80	F	Rectum		1	Mucinous adenocarcinoma
18	66	F	Rectum		3	Signet ring cell carcinoma
19	87	F	Colon	Ascending	3	Medullary carcinoma
20	41	M	Colon	Descending	2	Mucinous adenocarcinoma
21	65	F	Colon	Ascending	1	Serrated adenocarcinoma
22	65	M	Colon	Ascending	3	Signet ring cell carcinoma
23	74	M	Colon	Ascending	1	Serrated adenocarcinoma
24	57	M	Rectum		1	Serrated adenocarcinoma
25	77	M	Colon	Ascending	3	Adenocarcinoma
26	55	F	Colon	Transverse	2	Adenocarcinoma
27	54	F	Rectum		1	Adenocarcinoma
28	67	M	Colon	Ascending	2	Mucinous adenocarcinoma
29	61	M	Colon	Descending	2	Adenocarcinoma
30	52	F	Colon	Ascending	3	Signet ring cell carcinoma
31	37	F	Colon	Sigmoid	3	Signet ring cell carcinoma
32	56	F	Colon	Transverse	1	Signet ring cell carcinoma
33	46	F	Colon	Transverse	3	Mucinous adenocarcinoma

(F: female; M: male; 1: well-differentiated, grade I; 2: moderately differentiated, grade II; 3: poorly differentiated, grade III)

**Table 2.** Classification of the samples used according to the TNM system with their respective stage.

Code	T	N	M	TNM
1	T4a	N0	M1a	IVA
2	T3	N0	M0	IIA
3	T4a	N0	M1a	IVA
4	T4a	N0	M0	IIB
5	T3	N0	M1a	IVA
6	T3	N0	M1a	IVA
7	T3	N0	M0	IIA
8	T3	N2a	M0	IIIB
9	T4a	N0	M1a	IVA
10	T2	N2b	M1a	IVA
11	T4a	N2b	M1a	IVA
12	T3	N2b	M0	IIIC
13	T3	N0	M0	IIA
14	T3	N0	M0	IIA
15	T3	N1a	M1a	IVA
16	T4a	N1b	M0	IIIB
17	T1	N0	M1a	IVA
18	T3	N0	M1a	IVA
19	T4a	N0	M1a	IVA
20	T4b	N0	M0	IIC
21	T3	N1a	M0	IIIB
22	T3	N0	M0	IIA
23	T1	N2a	M0	IIIA
24	T4b	N0	M1a	IVA
25	T3	N2a	M0	IIIC
26	T4a	N2a	M1a	IVA
27	T2	N0	M0	I
28	T3	N0	M0	IIA
29	T3	N2	M1a	IVA
30	T2	N0	M1a	IVA
31	T4a	N1a	M0	IIIB
32	T2	N1	M1a	IVA
33	T4a	N1	M1a	IVA

(T: Primary tumor; N: Regional lymphatic nodes; M: Distant metastasis)

**Table 3.** Results obtained from the analysis of the KRAS and NRAS genes by the SNaPshot method.

Code	KRAS	NRAS
1	WT	WT
2	WT	WT
3	c.34G>C p.Gly12Arg	WT
4	WT	WT
5	c.35G>T p.Gly12Val	WT
6	WT	c.35G>A p.Gly12Asp
7	WT	WT
8	c.37G>A p.Gly13Ser	WT
9	c.34G>C p.Gly12Arg	WT
10	WT	WT
11	WT	WT
12	WT	WT
13	WT	WT
14	WT	c.35G>A p.Gly12Asp
15	WT	WT
16	WT	WT
17	WT	WT
18	WT	WT
19	WT	WT
20	c.35G>A p.Gly12Asp	WT
21	WT	WT
22	WT	c.38G>C p.Gly13Ala
23	WT	c.34G>T p.Gly12Cys
24	WT	WT
25	WT	WT
26	WT	WT
27	WT	WT
28	c.35G>T p.Gly12Val	WT
29	c.35G>A p.Gly12Asp	WT
30	WT	WT
31	c.37G>A p.Gly13Ser	WT
32	c.35G>A p.Gly12Asp	WT
33	WT	c.35G>A p.Gly12Asp

**Table 4.** Distribution of KRAS mutations detected by SNaPshot.

Nucleotide change	Mutated protein	Number of samples	Relative frequency %	Absolute frequency%
Codon 12				
c.35G>A	p.Gly12Asp	3	33.3	9.1
c.35G>T	p.Gly12Val	2	22.2	6.1
c.34G>C	p.Gly12Arg	2	22.2	6.1
Codon 13				
c.37G>A	p.Gly13Ser	2	22.2	6.1
Total		9	100.0	27.3

(A: adenine; C: cytosine; G: guanine; T: thymine; Gly: glycine; Asp: aspartic acid; Val: valine; Arg: arginine, Ser: serine)

**Table 5.** Distribution of NRAS mutations detected by SNaPshot.

Nucleotide change	Mutated protein	Number of samples	Relative frequency%		Absolute frequency %
Codon 12					
c.35G>A	p.Gly12Asp	3		60.0	9.1
c.34G>T	p.Gly12Cys	1		20.0	3.0
Codon 13					
c.38G>C	p.Gly13Ala	1		20.0	3.0
Total		5		100.0	15.1

(A: adenine; C: cytosine; G: guanine; T: thymine; Gly: glycine; Asp: aspartic acid, Cys: cysteine, Ala: alanine)

**Table 6.** Diagnosis of mutations in the KRAS gene through the SNaPshot method and Sanger’s method.

Code	SNaPshot diagnostics	Sanger diagnostics
1	WT	c.34G>C p.Gly12Arg
2	WT	WT
3	c.34G>C p.Gly12Arg	WT
4	WT	WT
5	c.34G>C p.Gly12Arg	c.34G>C p.Gly12Arg
6	WT	WT
7	WT	WT
8	c.37G>A p.Gly13Ser	c.37G>A p.Gly13Ser
9	c.34G>C p.Gly12Arg	WT
10	WT	WT
11	WT	WT
12	WT	WT
13	WT	WT
14	WT	WT
15	WT	WT
16	WT	WT
17	WT	WT
18	WT	WT
19	WT	WT
20	c.35G>A p.Gly12Asp	c.35G>A p.Gly12Asp
21	WT	WT
22	WT	WT
23	WT	WT
24	WT	WT
25	WT	WT
26	WT	WT
27	WT	WT
28	c.35G>T p.Gly12Val	c.35G>T p.Gly12Val
29	c.35G>A p.Gly12Asp	c.35G>A p.Gly12Asp
30	WT	WT
31	c.37G>A p.Gly13Ser	c.37G>A p.Gly13Ser
32	c.35G>A p.Gly12Asp	c.35G>A p.Gly12Asp
33	WT	WT

**Table 7.** Obtained sensitivity and specificity values for the SNaPshot and Sanger methods.

	SNaPshot	Sanger
*Sensitivity (%)*	90.0	77.8
*Specificity (%)*	100.0	95.8
